# Placebo Effects on the Immune Response in Humans: The Role of Learning and Expectation

**DOI:** 10.1371/journal.pone.0049477

**Published:** 2012-11-21

**Authors:** Antje Albring, Laura Wendt, Sven Benson, Oliver Witzke, Andreas Kribben, Harald Engler, Manfred Schedlowski

**Affiliations:** 1 Institute of Medical Psychology and Behavioral Immunobiology, University Hospital Essen, Essen, Germany; 2 Department of Nephrology, University of Duisburg-Essen, Essen, Germany; Tokai University, Japan

## Abstract

Placebo responses are primarily mediated via two neuropsychological mechanisms: patients’ expectation towards the benefit of a treatment and associative learning processes. Immune functions, like other physiological responses, can be modulated through behavioral conditioning. However, it is unknown whether learned immune responses are affected by the number of re-expositions to the conditioned stimulus (CS) during evocation. Moreover, it is unclear whether immune functions can also be modulated through mere verbally induced expectation. In the experiments reported here, we investigated in healthy male volunteers with an established model of learned immunosuppression whether a single re-exposition to the CS is able to induce a behaviorally conditioned immunosuppression. This conditioned immunosuppression is reflected through a significantly decreased interleukin (IL)-2 production by anti-CD3 stimulated peripheral blood mononuclear cells. Our data revealed that in contrast to four CS re-expositions (control group n = 15; experimental group n = 17), a single CS re-exposition was not sufficient to significantly suppress IL-2 production (control group n = 9, experimental group n = 10). Furthermore, we could demonstrate that mere expectation of taking an immunosuppressant did not cause an immunosuppressive response (n = 8–9 per expectation condition). Together, these findings extend our knowledge about the kinetics and mechanisms of placebo-induced immunosuppression and provide therewith information for designing conditioning protocols, which might be employed as a supportive therapy in clinical settings.

## Introduction

A placebo is defined as a sham drug or treatment inducing positive effects caused by nonspecific treatment ingredients. Placebo responses have been intensely investigated in the field of pain [1], [2], where placebos have been shown to activate the endogenous opioid system [3], [4]. Furthermore, placebo treatment positively affects the symptoms and clinical course of different diseases such as Parkinsońs disease [5] or asthma [6], [7]. Two distinct but interrelated neuropsychological mechanisms seem to play a central role steering the placebo response: expectation of patients or subjects towards the benefit of a forthcoming treatment and associative learning processes [8]. However, there is only sparse knowledge about which of these two factors is mediating the placebo response in various clinical or experimental conditions. Numerous studies meanwhile document that expectation is mediating placebo responses in many clinical conditions such as pain or Parkinson disease [9], [10], [11]. In Parkinson patients significantly increased dopamine release and motor performance were observed when expectation was induced to receive an active medication [11], [12]. Positive expectation was demonstrated to enhance the analgesic effect of a drug while negative expectation in form of an increased experience of pain sensation abrogated the drug effect [13].

Additionally, it has been shown that expectation induced placebo analgesia can be maximized through prior exposure to an effective therapy emphasizing the important role of learning in the process generating the placebo response [14]. In contrast, peripheral physiological functions such as secretion of growth hormone and cortisol were not affected through mere manipulation of expectancy but through behavioral conditioning [11]. More recently, expectation-induced placebo responses improved the subjective well being in asthma patients, however did not affect the forced expiratory volume analyzed by spirometry [7]. Thus, whether and to what extend cognitive factors such as expectation or associative learning processes are affecting peripheral organ functioning is rather unclear so far.

Experimental evidence in rodents and humans demonstrates that immune cell functions can be modulated through behavioral conditioning [15], [16], [17], [18]. In a well-established conditioning paradigm in humans, the immunosuppressive drug cyclosporine A (CsA) (unconditioned stimulus/US) is paired with a gustatory stimulus (conditioned stimulus/CS) during acquisition. Mere re-exposition to the CS during evocation is mimicking the immunopharmacological properties of CsA, reflected by impaired Th1 cytokine production and decreased T cell proliferation [18], [19]. It is unclear however, whether the extent of the learned immunosuppression in humans is depending on the number of US-CS pairings or the number of CS re-expositions as previously shown in rodents [20]. Furthermore, it is completely unknown whether the immunosuppressive effects can be also induced through mere expectation of receiving an immunosuppressive drug.

Therefore, using an established conditioning paradigm in healthy volunteers, the present study aimed to investigate firstly, whether the learned immunosuppression is affected by the number of CS re-expositions (experiments A and B) and secondly, whether a suppression of T cell functions can be achieved by mere verbally induced expectation of receiving the immunosuppressive drug CsA (experiment C).

## Materials and Methods

### Ethics Statement

The study was approved by the local ethics committee for human investigations of the University Hospital Essen and follows the rules stated in the Declaration of Helsinki. All participants gave written informed consent and were reimbursed for their participation.

### Subjects

Healthy male volunteers (age range: 18–40 years) were recruited through public advertisement in the surrounding community. All volunteers underwent an intense physical and psychiatric assessment (self-reported questionnaires, interview about the medical history) and were in addition subjected to an electrocardiogram and ultrasonography of the kidneys, evaluated by the physicians of the Department of Nephrology. Subjects were excluded if one of the following criteria was identified: daily intake of medication, blood donations>200 ml within the last two months, intolerance (e.g. lactose intolerance) for substances used in the study, previous participation in pharmacological studies or other medical exclusion criteria (e.g. disorders of immune or endocrine system, previous or persistent psychiatric disorders, allergies, signs of cardiovascular, hematologic or nephrologic disorders, respiratory problems, addiction or diabetes mellitus). After inclusion in the study, participants were randomly allocated to control and experimental groups.

### Experimental Protocols

#### Behavioral conditioning

**Figure 1:**
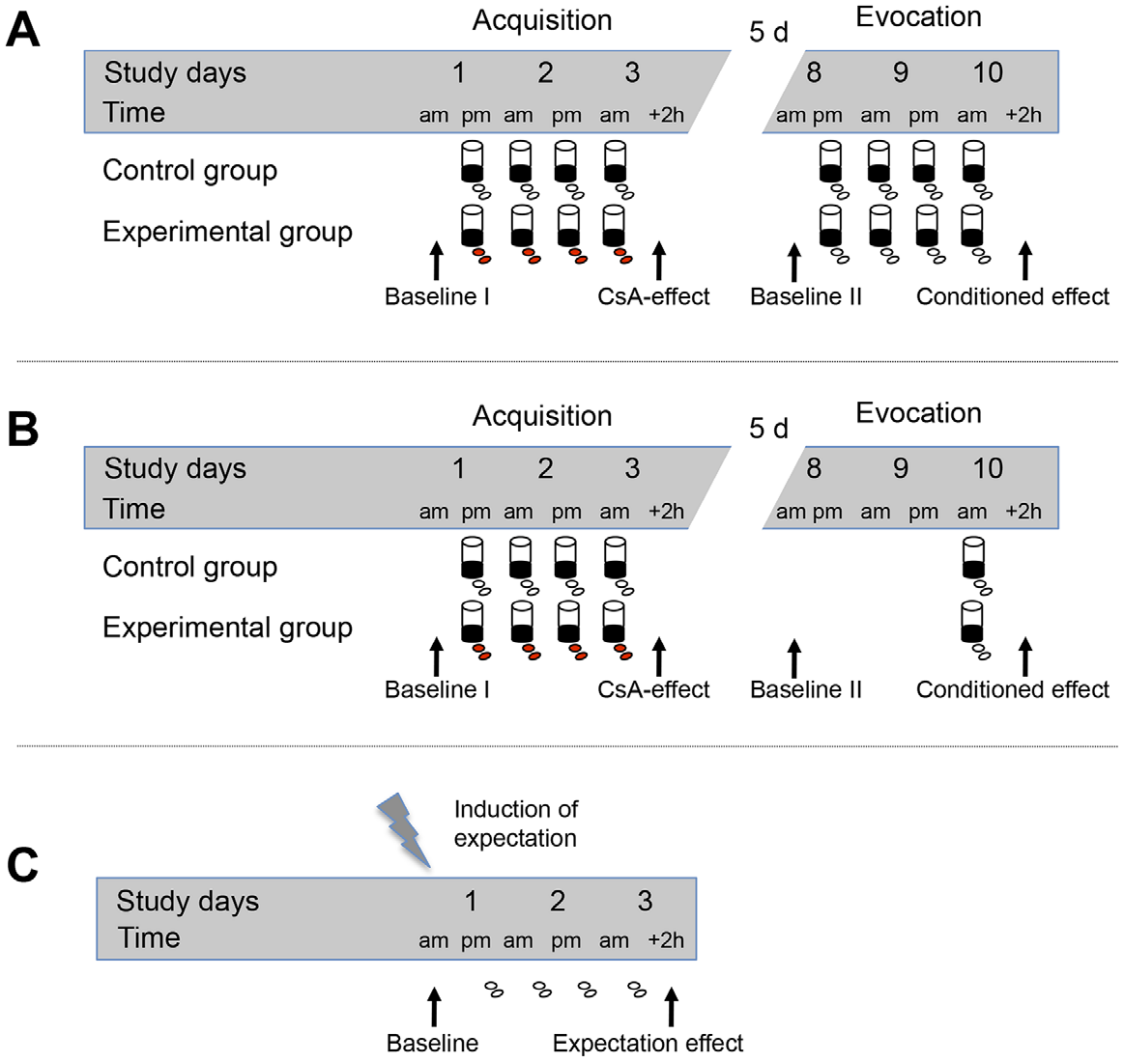
Experimental design. (A) During the acquisition phase in conditioning *experiment A*, subjects of the experimental group received four times cyclosporin A (CsA) as an US together with a green-colored, novel tasting drink, the CS. During evocation, subjects were re-exposed to the drink four times but received identically looking placebo capsules instead of CsA. The control group was treated in an identical way but received placebo capsules throughout the study. Blood was drawn on the first day (baseline), on day 3 to determine the CsA-effect, on day 8 to analyze possible residual drug effects and on day 10 in order to determine the conditioned effect on IL-2 production [19]. (B) During the acquisition phase in conditioning *experiment B* subjects were identically treated as in *experiment A*. However, during evocation, subjects were re-exposed to the drink and the placebo capsules only once. Blood was drawn on the first day (baseline), on day 3 to determine the CsA-effect, on day 8 to analyze possible residual drug effects and on day 10 in order to determine the conditioned effect on IL-2 production. (C) In *experiment C*, subjects were told to have a probability of either 25%, 50%, 75% or 100% of receiving CsA to manipulate subjects’ expectation of receiving an active drug. Capsules were given at four time points on 3 consecutive days. Blood was drawn on the first day for baseline measurement and on day 3 to determine the potential effect of expectation on IL-2 production of anti-CD3 stimulated PBMC.

This study consists of two separate experiments (A and B) with almost identical experimental designs, except for the number of re-expositions to the conditioned stimulus during the evocation phase (Fig. 1A and 1B).

Experiment A: Thirty-two subjects (mean age: 25.7±4.2 years) participated in the double-blind placebo-controlled *experiment A* (Fig. 1A). Volunteers were randomly allocated to control (n = 15) and experimental groups (n = 17). During the acquisition phase subjects of the experimental group received on day 1 (6 pm), day 2 (8 am and 6 pm), and day 3 (8 am) oral doses of 2.5 mg/kg CsA (Sandimmun®, Novartis) in capsule form as an US together with a green-colored, novel-tasting drink (150 ml strawberry milk aromatized with lavender oil) as CS. Following a five day wash out period, subjects were re-exposed to the drink four times during the evocation phase (day 8 at 6 pm; day 9 at 8 am and 6 pm; day 9 at 8 pm) but instead of CsA they received identically looking placebo capsules. This behavioral protocol was based on our previous experience with behavioral conditioning in humans and has been shown to induce a conditioned immunosuppression [18]. The control group (n = 15) was treated similarly but received placebo capsules throughout the study. Blood was drawn on the first day at 8 am (baseline) and on day 3 at 10 am to determine the pharmacological effects of CsA. Additionally, blood was drawn at 8 am on day 8 to analyze possible residual effects of the drug and at 10 am on day 10 in order to analyze behaviorally conditioned immunosuppressive responses after evocation (Fig. 1A). Participants were told that the chance of receiving CsA was always 50%. The data of *experiment A* (4 CS re-expositions) have been previously published presenting the immunological results as absolute IL-2 levels (pg/ml) [19]. However, for the present analyses these data have been re-calculated as percental changes from baseline to allow a direct comparison with data of *experiment B* (1 CS re-exposition).

Experiment B: In order to analyze whether the number of CS-re-expositions during evocation affects the magnitude of the learned immunosuppressive response subjects in *experiment B* (Fig. 1B) received only a single re-exposition to the CS in contrast to *experiment A* where four CS re-expositions during evocation were employed. Apart from the number of CS-re-expositions during evocation, *experiment B* was designed and performed identically to *experiment A* (19). Nineteen subjects (mean age: 26.9±0.9 years) were included in the double-blind placebo-controlled *experiment B.* Again subjects were randomly assigned to control (n = 9) and experimental groups (n = 10). Identically to *experiment A*, subjects of the experimental group received four times the CS paired with the US during the acquisition phase. However, in contrast to *experiment A*, subjects were re-exposed to the taste stimulus (CS) and the identically looking placebo capsules only once on day 10 (8 am) during evocation. The control group was treated similarly but received placebo capsules throughout the study. Blood was drawn at the same time points as in *experiment A* (day 1 at 8 am, day 3 at 10, day 8 at 8 am, day 10 at 10 am) (Fig. 1B). Participants were told that the chance of receiving CsA was always 50%.

#### Manipulation of expectation

In *experiment C*, verbal suggestions were employed to modulate the expectancy of 33 healthy male volunteers (mean age: 25.4±0.9 years). Subjects were told to have a probability of either 25% (n = 9), 50% (n = 8), 75% (n = 8), or 100% (n = 8) of receiving CsA to manipulate the perceived likelihood of taking an immunosuppressive drug. On day 1 at 8 am subjects drew a ticket, which assigned them to one of the four groups. The same day at 6 pm subjects had to choose one of four tablet boxes. Depending on the group, subjects were told that one (25% group), two (50% group), three (75% group) or all (100% group) of the four tablet boxes contain CsA-capsules. In fact, subjects never received active medication but placebo capsules throughout the study. Capsules were given at four time points, on day 1 at 6 pm, day 2 at 8 am and 6 pm and on day 3 at 8 am. Each time, subjects were informed about the immunosuppressive effects of CsA-treatment. Blood was drawn and cardiovascular parameters were measured on the first day at 8 am for baseline measurement and at 10 am on day 3 (Fig. 1C) to determine the potential effect of expectation on immunological variables.

### Cell Isolation

Peripheral blood mononuclear cells (PBMC) were isolated by density gradient centrifugation (Ficoll-Paque™ Plus, GE Healthcare, Munich, Germany). Cells were washed with Hanks’ Balanced Salt Solution (Life Technologies, Darmstadt, Germany), counted with an automated hematology analyzer (KX-21 N, Sysmex Deutschland GmbH, Norderstedt, Germany) and adjusted to 5×10^6^ and 2,5×10^6^ cells/ml in cell culture medium (RPMI 1640 supplemented with GlutaMAX I, 25 mM Hepes, 10% fetal bovine serum, 50 µg/ml gentamicin; Life Technologies).

### T cell Stimulation and Determination of IL-2 in Culture Supernatant

PBMC suspensions (100 µl; 5×10^6^ cells/ml) were transferred to 96-well flat bottom tissue culture plates and were stimulated with 20 ng/ml of soluble mouse anti-human CD3 monoclonal antibody (clone: HIT3a; BD Pharmingen, San Diego, CA) for 24 h (37°C, 5% CO_2_). Concentration of IL-2 in culture supernatants was quantified using a commercial bead-based assay (Bio-Plex Pro Human Cytokine Assays, Bio-Rad Laboratories, Hercules, CA) as previously described [19], [21] according to the manufacturers’ instructions. Briefly, sample dilutions were incubated with fluorescent beads conjugated to anti-human IL-2 antibodies. After incubation with IL-2 specific secondary antibodies and streptavidin-PE, samples were analyzed on a FACS Canto II flow cytometer using FACS Diva 6.01 software (BD Immunocytometry Systems, Heidelberg, Germany). Absolute IL-2 concentrations were calculated based on the mean fluorescence intensity of cytokine standards. The limit of detection was 1.4 pg/ml. The intra-assay variance was<10.8% and the inter-assay variance adds up to<12.5%.

### Intracellular Cytokine Staining

Intracellular IL-2 of activated CD4^+^T cells was detected by flow cytometry using Intracellular Cytokine Staining Starter Kit – Human (BD Pharmingen). PBMC (1 ml; 2.5×10^6^ cells/ml) were incubated for 3.5 h (37°C, 5% CO_2_) with 2 µl Leukocyte Activation Cocktail (BD Pharmingen), containing PMA, ionomycin and the protein transport inhibitor brefeldin A. Cells were double-stained with PE-Cy7 conjugated anti-human CD3 (clone SK7; BD Pharmingen) and APC conjugated anti-human CD4 (clone RPA-T4, BD Pharmingen) antibodies to characterize CD4^+^T cells. Cells were fixed and permeabilized using Cytofix/Cytoperm Buffer containing a mixture of paraformaldehyde and saponin. Perm/Wash Buffer maintains the cellular permeability and was used for washing- and intracellular staining steps. PE conjugated anti-human IL-2 (clone MQ1-17H12, BD Pharmingen) antibody was used for intracellular cytokine staining. The percentage of IL-2 producing CD4^+^T cells was analyzed on a FACS Canto II flow cytometer using FACS Diva 6.01 software (BD Immunocytometry Systems, Heidelberg, Germany).

### Behavioral Measures

Sociodemographical data were collected from all participants. Subjects also completed the state version of the State-Trait-Anxiety-Inventory (STAI) [22] and Beck Depression Inventory scores (BDI) [23] in order to document possible group differences in present state negative emotions (STAI) and depressive symptoms.

### Statistical Analysis

All data are expressed as mean ± SEM. The Kolmogorov-Smirnov-test was used to determine whether the data met the assumption of normality. For non-normally distributed variables (i.e., cytokine data in experiments A and B), logarithmic transformations were applied prior to data analysis. Immunological parameters were compared with unpaired *t*-tests (experiments A and B) and repeated measures analysis of variance (ANOVA) (experiment C). Sociodemographical and psychological characteristics were analyzed with univariate ANOVA or chi^2^-tests. The significance-level was set at *p<*0.05. Data were analyzed using PASW statistics (version 18, SPSS, Chicago, IL, USA).

## Results

### Behavioral Conditioning

**Figure 2:**
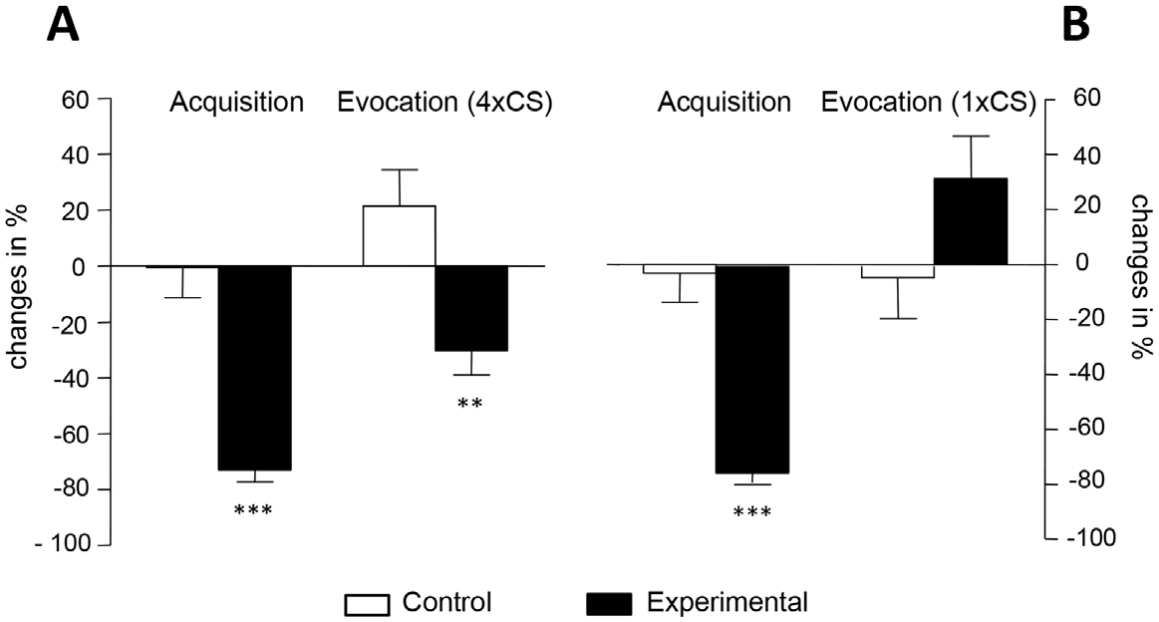
Behavioral conditioning induced cytokine response. Behaviorally conditioned suppression of IL-2 release was observed after four CS re-expositions (control group n = 15, experimental group n = 17) (Fig. 2A). In contrast, a single CS re-exposition did not induce a significant inhibition in IL-2 production (control group n = 9, experimental group n = 10) (Fig. 2B). Data are expressed as percental changes from baseline. Bars represent mean ± SEM; ** *p*<0.01, *** *p*<0.001.

Subjects of the experimental group of *experiment A* and *experiment B* did not significantly differ from subjects of the respective control groups in sociodemographic and screening variables, i.e., age, body mass index, smoking behavior, Beck Depression Inventory scores and in behavioral trait anxiety variables (Table S1). IL-2 levels in culture supernatants of anti-CD3 stimulated PBMC were determined to analyze whether the extent of the learned immunosuppression depends on the number of CS re-expositions. Administration of CsA during the acquisition phase significantly suppressed the IL-2 production *(p*<0.001) in *experiment A* (*t* = 7.1; *p*<0.001) as well as in *experiment B* (*t* = 6.8; *p*<0.001) (Fig. 2A, 2B). As previously published [18], [19], [24] four re-expositions to the CS during the evocation phase induced a significant behaviorally conditioned reduction in IL-2 release from anti-CD3 stimulated PBMC (*t* = 3.9; *p<*0.01), shown here as percental changes from baseline (Fig. 2A). In contrast, a single re-exposition to the CS during the evocation phase (*experiment B*) was not sufficient to suppress IL-2 release from activated PBMC in comparison to the respective control group (*t* = −1.5; n.s.) (Fig. 2B).

### Manipulation of Expectation

**Figure 3:**
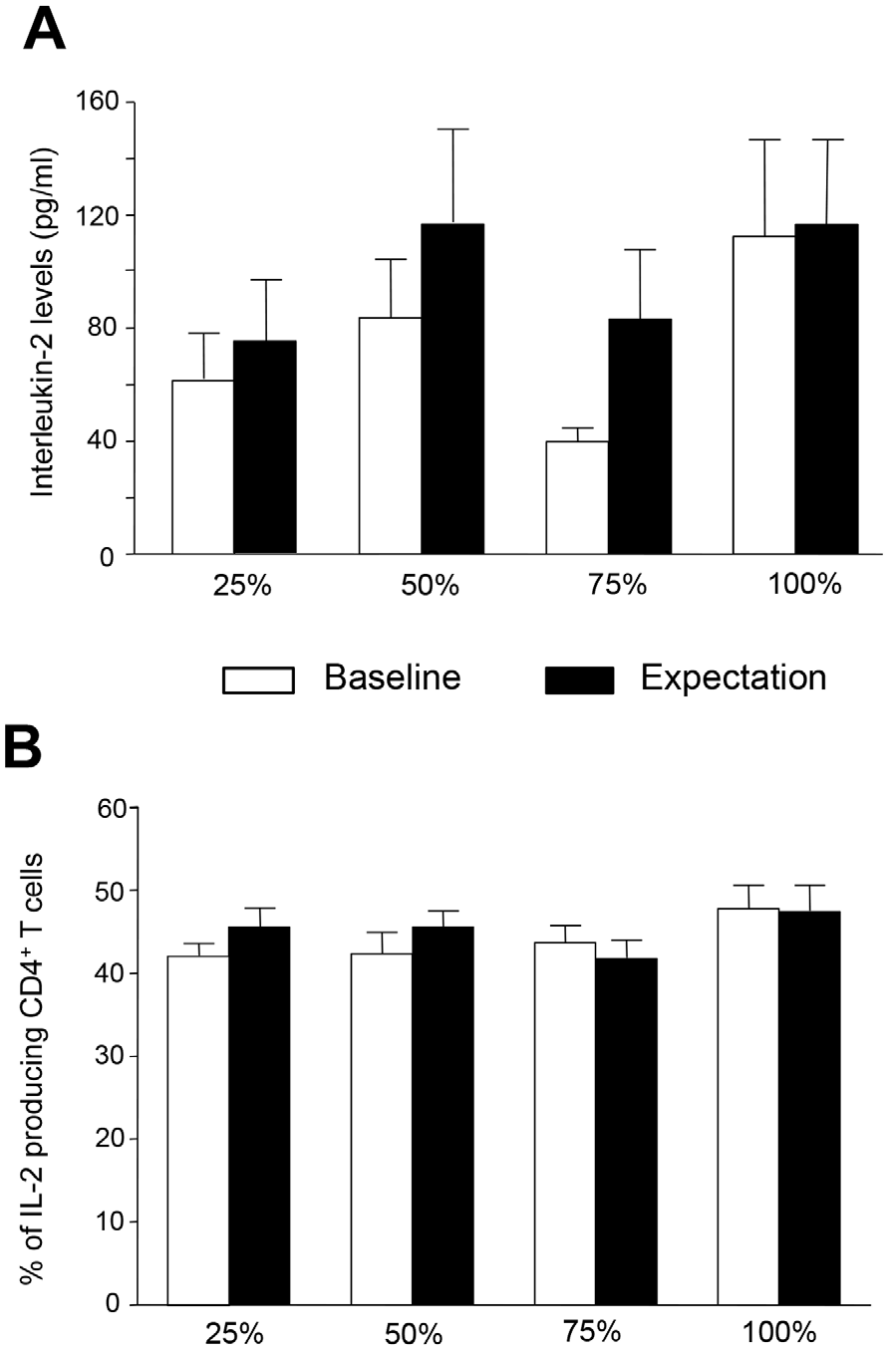
Expectation induced cytokine response. (A) Expectation did not induce a significant reduction in IL-2 production in any of the four expectation groups differing in the probability of receiving CsA. IL-2 (pg/ml) concentration in supernatants of anti-CD3 stimulated PBMC was analyzed before and after placebo pill intake (25% n = 9, 50% n = 8, 75% n = 8, 100% n = 8). Data are shown as mean ± SEM. (B) Expectation did not reduce the percentage of IL-2 producing CD4^+^T cells. IL-2 producing CD4^+^T cells were analyzed as percent of total CD4^+^T cells before and after placebo pill intake. Data are shown as mean ± SEM.

In *experiment C*, subjects were randomly allocated to four groups differing in the suggested probability (25%, 50%, 75%, 100%) of receiving the immunosuppressive drug CsA. Subjects of the four expectation groups did not differ in sociodemographic and screening variables, i.e., age, body mass index, smoking behavior, Beck Depression Inventory scores and in behavioral trait anxiety variables. Furthermore, groups did not significantly differ in cardiovascular parameters before and after induced expectation (Table S2). IL-2 levels in culture supernatants of anti-CD3 stimulated PBMC were analyzed before (baseline) and after intake of the placebo pills (expectation effect) to determine the effect of different expectations on IL-2 release. The expectation of receiving an immunosuppressive drug did not significantly affect IL-2 secretion in any of the 4 probability-groups (ANOVA; group effect, *F* = 1.2; *p* = 0.33; interaction effect, *F* = 1.1; *p* = 0.35) (Fig. 3A). As an additional parameter, the percentage of IL-2 producing PMA/Ionomycin-stimulated CD4^+^T cells was analyzed before and after the pill intake by intracellular cytokine staining. Again, these results did not show a significant reduction of IL-2 producing CD4^+^T cells in any of the four expectation groups compared to the control condition during baseline (ANOVA; group effect, *F* = 0.4; *p* = 0,732; interaction effect, *F* = 1.2; *p* = 0.334) (Fig. 3B).

## Discussion

The placebo response is generated by two distinct but interrelated mechanisms across different physiological systems and clinical conditions. One of these mechanisms concerns suggestion and expectation, the other one learning via behavioral conditioning. While for example placebo analgesia is mediated through cognitive factors as well as learning procedures it still remains unclear whether autonomous functions such as secretion of endocrine factors or immune responses can only be manipulated through learning processes or also via mere expectation. The results presented here demonstrate that immunosuppressive effects, reflected by a significant inhibition of IL-2 release by anti-CD3 stimulated PBMC, can be induced via repeated [19], however not through a single re-exposition to the CS during evocation. Furthermore, the data reveal that mere verbally induced expectation of receiving the immunosuppressive drug CsA did not significantly decrease IL-2 secretion of activated PBMC, regardless of the declared probability of receiving active medication. Thus, our data support and extend previous observations that autonomous physiological functions can be influenced via behavioral conditioning processes but not through mere manipulation of cognitive factors [11].

In Parkinson disease, mere expectation of therapeutic benefit has been repeatedly shown to be associated with endogenous dopamine release [25], [26], [27]. More recently, dopamine secretion in Parkinson patients was demonstrated to significantly increase when the declared probability of receiving an active treatment was 75%. In contrast, the conditions with a stated probability of 25%, 50% or 100% did not affect central dopamine release. These data reveal that the perceived likelihood of receiving an active treatment directly modulates the placebo response in Parkinson patients, suggesting that the dopaminergic system is activated when the therapeutic benefit is likely but not certain [12]. On the basis of this experimental setting we designed four experimental groups, differing in the declared probability (25%, 50%, 75% or 100%) of receiving the immunosuppressant CsA and analyzed the effect of expectation on IL-2 release by anti-CD3 stimulated PBMC. However, we did not observe an expectation-induced effect on IL-2 production in any of the four probability groups, suggesting that peripheral immune functions cannot be affected through mere cognitive factors. Thus, it appears that scientific findings concerning the experimental conditions under which placebo responses occur cannot be generalized and easily transferred from one disease or system to another [11], [28]. Even the results of studies focusing on the same disease but differing in study design or read out parameters are partly contradictory and difficult to compare [7]. In asthma patients a placebo bronchodilator significantly reduced nonspecific airway hyper-responsiveness, suggesting that expectation-induced placebo responses are not confined to subjective symptoms but also influence objective outcomes [6]. However, a recent study with asthma patients contradicts these findings documenting that mere expectation of therapeutic benefit ameliorates the subjective symptoms but does not significantly improve objective lung function indicating that peripheral organ functions cannot be affected through mere cognitive factors [7]. Regarding the allergic reactions of patients suffering from allergic house-dust-mite rhinitis it has been demonstrated that the anti-histaminergic properties of the H_1_-receptor antagonist desloratadine can be behaviorally conditioned, as analyzed by subjective symptom score, skin prick test and decreased basophile activation. Interestingly, subjective symptom score and skin reactivity, but not basophile activation, were reduced in patients who where conditioned but not re-exposed to the CS but to water while conditioned patients who were re-exposed to the CS also demonstrated significantly decreased basophile activation [29]. These data re-emphasize the important role of learning as source of the placebo response when autonomous physiological functions such as endocrine or immune responses are concerned.

The potential therapeutic relevance of learned immune responses has been impressively documented in experimental models for chronic inflammatory autoimmune diseases [30], [31], tumor development [32], [33], or organ transplantation [34], [35] where a learned immunosuppression decreased disease exacerbation and mortality. These data highlight the clinical significance of conditioning paradigms in settings where an inhibition of immune functions is required [8], [17], [36]. However, before these learning protocols can be considered as a treatment option to support a pharmacological regimen, detailed knowledge about the kinetics, reproducibility and most effective use of those paradigms is urgently needed. Therefore, regarding the kinetics of learned immunosuppression, the present study analyzed whether a learned immunosuppression can be detected not only after four re-expositions to the CS [19] but also already after a single CS re-exposure. Our data clearly demonstrate that a single re-exposition to the CS is not sufficient to induce an immunosuppressive response. This finding confirms previous data of animal studies revealing that once consolidated, the extinction of the taste-CsA engram is prolonged and the more this engram is activated at evocation, the more pronounced is the behaviorally conditioned immunosuppression [20].

We could recently demonstrate in both rodents and humans that the learned immunosuppression is not restricted to a single event but is retrievable and can be repeatedly recalled [19]. In addition, plasma noradrenaline and state anxiety seem to predict this learned immunosuppression in placebo responders [24]. Regarding these findings it is important to consider that repeated unreinforced re-expositions to the CS will finally lead to an extinction of the conditioned response [37]. However, the processes modulating extinction learning in conditioned immunosuppression such as context changes, retention intervals and reconsolidation are for the most part unknown [38]. Thus, regarding the potential feasibility of the conditioning procedure in clinical situations, further research focusing on the question how to overcome the extinction process is of essential significance.

Furthermore, a better understanding of the neurobiological mechanisms steering learned immune responses is of great importance. So far, experimental evidence reveals that the learned immune response employing CsA as an US is mediated centrally via the insular cortex, the amygdala and the ventromedial nucleus of the hypothalamus [39] and steered via sympathetic innervation of the spleen, noradrenaline and β-adrenoreceptor-dependent mechanisms [40]. However, more detailed knowledge about the kinetics of the learned immune response and the mechanisms behind the CNS-immune system interaction is urgently needed. Only with this information it will be possible to design conditioning protocols which can be employed in clinical settings to reduce the required drug dose while maximizing the therapeutic outcome for the patients benefit [8].

## Supporting Information

Table S1 **Sociodemographic and psychological characteristics (experiment A and B).** No significant differences between experimental and respective control groups were observed in experiments A and B (results of unpaired samples t-tests or chi^2^-test, all p>0.05)
(DOCX)

Table S2 **Sociodemographic and psychological characteristics and cardiovascular parameters (experiment C).** Age, body mass index, Beck Depression Inventory and trait anxiety (STAI) scores were compared between the four experimental groups using univariate analysis of variances (ANOVA), as well as smoking behaviour (by chi^2^-test). Changes in cardiovascular parameters (i.e., heart rate, systolic blood pressure, diastolic blood pressure) were analyzed in the four groups before and after induction of expectation (ANOVA group × time interaction). No significant group or interaction effects were observed (all p>0.05).Data are shown as mean± SEM.
(DOCX)
