# Supplementary material for: Placebo Effects on the Immune Response in Humans: The Role of Learning and Expectation
Source: PLoS One. 2012 Nov 21;7(11):e49477. doi: 10.1371/journal.pone.0049477 (PMC3504052; doi:10.1371/journal.pone.0049477)
Supplement: Table S1 — Sociodemographic and psychological characteristics (experiment A and B). No significant differences between experimental and respective control groups were observed in experiments A and B (results of unpaired samples t-tests or chi2-test, all p>0.05) (DOCX) [file pone.0049477.s001.docx]

**Table S1: Sociodemographic and psychological characteristics (experiment A and B)**

|  | experimental group (experiment A) n=17 | control group (experiment A) n=15 | experimental group (experiment B) n=10 | control group (experiment B) =9 |
| --- | --- | --- | --- | --- |
| Age, years | 25.4± 1,0 | 26,4± 1,2 | 27± 1,0 | 27,0± 1,7 |
| Body mass index (kg/m²) | 23± 0,4 | 23,9± 0,6 | 21.3± 2,5 | 21,9± 2,9 |
| Smoking behavior | 29,4% smokers | 20% smokers | 40% smokers | 33,3% smokers |
| Beck depression Inventory scores | 3,9± 0,9 | 4,2± 0,8 | 4,7± 0,8 | 3,7± 1,1 |
| Trait anxiety (STAI) | 34,06± 1,8 | 31,4± 1,6 | 38,4± 2,3 | 31,0± 4,4 |

No significant differences between experimental and respective control groups were observed in experiments A and B (results of unpaired samples t-tests or chi²-test, all p> 0.05)
