# Supplementary material for: Placebo Effects on the Immune Response in Humans: The Role of Learning and Expectation
Source: PLoS One. 2012 Nov 21;7(11):e49477. doi: 10.1371/journal.pone.0049477 (PMC3504052; doi:10.1371/journal.pone.0049477)
Supplement: Table S2 — Sociodemographic and psychological characteristics and cardiovascular parameters (experiment C). Age, body mass index, Beck Depression Inventory and trait anxiety (STAI) scores were compared between the four experimental groups using univariate analysis of variances (ANOVA), as well as smoking behaviour (by chi2-test). Changes in cardiovascular parameters (i.e., heart rate, systolic blood pressure, diastolic blood pressure) were analyzed in the four groups before and after induction of expectation (ANOVA group × time interaction). No significant group or interaction effects were observed (all p>0.05).Data are shown as mean± SEM. (DOCX) [file pone.0049477.s002.docx]

**Table S2: Sociodemographic and psychological characteristics and cardiovascular parameters (experiment C)**

|  | **25% expectation n=9** | | **50% expectation n=8** | | **75% expectation n=8** | | **100% expectation n=8** | |
| --- | --- | --- | --- | --- | --- | --- | --- | --- |
| Age, years | 25,56± 1,3 | | 23,38± 1,3 | | 26,50± 2,4 | | 26,25± 1,8 | |
| Body mass index (kg/m²) | 25,04± 1,7 | | 24.11± 0.9 | | 24,26± 1,2 | | 25, 52± 0,9 | |
| Smoking behavior | 44,4% smokers | | 12,5% smokers | | 12,5% smokers | | 12,5% smokers | |
| Beck depression Inventory scores | 4,6± 1,1 | | 2,3± 0,9 | | 4,9± 1,4 | | 5.5± 1,1 | |
| Trait anxiety (STAI) | 33,2± 2,6 | | 33± 2,5 | | 33,6± 2,3 | | 36,0± 1,4 | |
| **Cardiovascular parameters** | **before** | **after** | **before** | **after** | **before** | **after** | **before** | **after** |
| Heart rate (bpm) | 68± 2,4 | 62,7± 2,1 | 75,5± 3,7 | 67± 3,6 | 65,5± 4,3 | 67± 4,6 | 70± 3,6 | 66,5± 3,2 |
| Systolic blood pressure (mmHG) | 122,2± 4,0 | 122,2± 3,5 | 122,5± 3,1 | 117,5± 4,5 | 124,4± 5,3 | 119,4± 3,3 | 121,3± 2,8 | 117,5± 3,1 |
| Diastolic blood pressure (mmHG) | 81,7± 2,0 | 79,0± 2,2 | 80,6± 0,6 | 77,0± 3,4 | 82,5± 3,1 | 80,0± 1,9 | 76,3± 1,8 | 74,4± 1,8 |

Age, body mass index, Beck Depression Inventory and trait anxiety (STAI) scores were compared between the four experimental groups using univariate analysis of variances (ANOVA), as well as smoking behaviour (by chi²-test). Changes in cardiovascular parameters (i.e., heart rate, systolic blood pressure, diastolic blood pressure) were analyzed in the four groups before and after induction of expectation (ANOVA group x time interaction). No significant group or interaction effects were observed (all p> 0.05).Data are shown as mean± SEM.
